# Steinmann pin retractor: An auxiliary reduction equipment for the minimally invasive treatment of calcaneal fractures

**DOI:** 10.1097/MD.0000000000030847

**Published:** 2022-09-30

**Authors:** Bin Zhao, Wenqian Zhao, Chao Liu, Isaac Assan, Rongxiu Bi

**Affiliations:** a Postdoctoral Research Station, Shandong University of Traditional Chinese Medicine, Jinan, Shandong, China; b Department of Orthopedics, Shouguang Hospital of Traditional Chinese Medicine, Shouguang, Shandong, China; c Weifang Key Laboratory for the Prevention and Treatment of Geriatric Diseases, Shouguang, Shandong, China; d Department of Geriatric Medicine, Qilu Hospital, Cheeloo College of Medicine, Shandong University, Jinan, Shandong, China; e Department of Traditional Chinese Medicine, The People’s Hospital of Shouguang City, Shouguang, Shandong, China; f College of Medicine and Veterinary Medicine, Deanery of Molecular, Genetic and Population Health Sciences, The University of Edinburgh, Old College South Bridge, Edinburgh, UK; g Department of Orthopedics, Affiliated Hospital of Shandong University of Traditional Chinese Medicine, Jinan, Shandong, China.

**Keywords:** calcaneal fracture, sinus tarsi approach, Steinmann pin retractor

## Abstract

A minimally invasive approach has been widely used for the treatment of calcaneal fractures, however, its downside in exposing the calcaneal body, affects fracture reduction. We used a Steinmann pin retractor mentioned in our previous case series study to solve this issue. To further evaluate the efficacy of this reduction technique, and elucidate its mechanism, we compared it with the control groups in this study. Between March 2017 and November 2020, 52 patients were included in this retrospective comparative study. 20 patients were included in the S-S group (Steinmann pin retractor with internal fixation via the sinus tarsi approach), 17 patients were included in the S group (internal fixation via sinus tarsi approach), and 15 patients were included in the L group (internal fixation via L-shaped approach). Patients in each group were operated on by a distinctly skilled surgeon who is specialized in one of the specified surgeries mentioned above. All patients received positive postoperative radiological and clinical evaluations. The patients were followed up for at least 6 months postoperatively. At the last S-S group follow-up, the VAS value (0.29 ± 0.46) improved compared to the L group. The AOFAS (American Orthopedic Foot and Ankle Society) Ankle-Hindfoot score (94.38 ± 5.05) also improved significantly compared to the S and L groups. The Böhler angle (32.34 ± 3.56°), width (36.48 ± 3.91 mm), and height (88.87 ± 4.12 mm) of the calcaneal improved (34.38 ± 18.50°, –10.13 ± 6.98 mm, 8.75 ± 4.82 mm) compared to the preoperative state, the S and L groups. These parameters (the Böhler angle: 31.76 ± 3.37°, width: 36.47 ± 3.72 mm, height: 87.23 ± 3.83 mm) were maintained at the last follow-up. Steinmann pin retractor effected a sound reduction. It also manifested radiological and clinical advantages over the S and L protocols. By reason of the aforementioned, it could be recommended as useful reduction equipment for the minimally invasive treatment of calcaneal fractures.

## 1. Introduction

The minimal invasive approach represented by the sinus tarsi approach has been widely used in open reduction and internal fixation (ORIF) for intra-articular calcaneal fractures. Compared with the traditional L-shaped approach, the minimally invasive approach has lower incision complications^[1]^ and is convenient for exposing the posterior articular surface. However, it is unable to fully expose the calcaneal body, tuberosity, or sustentaculum tail.^[2]^ Directly reducing the fracture of the calcaneal body or tuberosity via the sinus tarsi approach is not easy. It requires a comprehensive knowledge of the anatomy of the calcaneus, and completion of the reduction with a “joystick” under C-arm fluoroscopy in a closed manner by a skilled surgeon.^[3]^ This is a real challenge for skilled surgeons, let alone the novice. Could there be an alternative way to reduce the fracture perfectly via a minimally invasive incision? Our answer to this, is the Steinmann pin retractor. It was referred for the treatment of calcaneus fractures in our previous case series.^[4]^

However, critical evaluations should be done to determine the advantages Steinmann pin retractor has over the closed manner with a “joystick” or traditional surgical procedure via the L approach, and the mechanism underlining the retractor must be well elucidated. To evaluate the efficacy of the Steinmann pin retractor via the sinus tarsi approach, we retrospectively compared it to the internal plate-screw system fixation via sinus tarsi (S, without retractor) and traditional L-shaped (L, without retractor) approach in this study.

## 2. Materials and Methods

### 2.1. Ethics approval and consent to participate

This study was approved by the Committee on Medical Ethnic of Shouguang Hospital of Traditional Chinese Medicine (Permit number: 20170301) and was carried out in strict accordance with the recommendations in the Guide of “*Methods for ethical review of biomedical research involving humans (2016*)” from the state health and family planning commission of the People’s Republic of China. All patients provided written, informed consent for the surgery. This study has been registered and the Unique Identifying number is researchregistry5092 (Supplemental Digital Content, http://links.lww.com/MD/H444).

### 2.2. Patients

Between March 2017 and November 2020, 52 patients who met the inclusion criteria but not the exclusion criteria were included in our retrospective comparative study. These were patients with intra-articular calcaneal fractures from the department of orthopedics of Shouguang Hospital of Traditional Chinese Medicine.

### 2.3. Inclusion criteria

Patients who had intra-articular calcaneal fractures (Sanders type Ⅱ or Ⅲ) with a displacement of more than 2 mm, and received ORIF were included. Patients with accompanied bilateral, lumber, or lower limb fracture that did not affect the calcaneal surgery procedure and clinical evaluation postoperatively were also included.

### 2.4. Exclusion criteria

This retrospective comparative study excluded single extra-articular fractures, Sanders type Ⅰ or Ⅳ fractures, and non-displaced intra-articular fractures. Patients with open fractures, non-plate-screw system fixation, and loss of follow-up were also excluded.

In this retrospective study, 20 patients were included in the S-S group (Steinmann pin retractor with plate-screw system fixation via the sinus tarsi approach), 17 patients were included in the S group (plate-screw system fixation via sinus tarsi approach), and 15 patients were included in the L group (plate-screw system fixation via L-shaped approach). The demographic and group information, including age, sex, affected side, injury mechanism, Essex-Lopresti type, Sanders type, accompanied fractures, plate-screw system, smoking history, the time from trauma to operation, operation duration, were presented in Table 1.

**Table 1:** Demographic data and clinical results of patients.

	S-S (20 patients)	S (17 patients)	L (15 patients)
Age(years)	45.5 ± 10.7	42.8 ± 11.4	50.4 ± 12.7
Sex			
Male (n)	20	16	14
Female (n)	0	1	1
Affected side			
Left (n)	13	9	10
Right (n)	8	9	7
Injury mechanism			
Felling from a height (n)	18	17	15
Traffic accident (n)	2	0	0
Essex-Lopresti type			
Tongue type (n)	15	7	9
Depression type (n)	6	11	8
Sanders type			
IIA (n)	7	6	3
IIB (n)	3	8	4
IIC (n)	0	0	2
IIIAB (n)	2	0	4
IIIAC (n)	8	3	2
IIIBC (n)	1	1	2
Accompanied fractures			
Lumber fracture (n)	1	0	1
Lower limb fracture (n)	0	1	1
Smoking history (n)	12(60%)	8(47.1%)	7(46.7%)
The time from trauma to operation (days)	4.4 ± 3.2	2.4 ± 2.4	3.8 ± 3.2
Operation duration (min)	70.0 ± 20.2	72.8 ± 16.7	70.0 ± 17.1
Plate-screw system			
Circular plate	21	4	0
2 or 3 arms straight plate	0	14	16
H straight plate	0	0	1
Follow-up time (months)	20.15 ± 6.26	16.82 ± 6.68	18.33 ± 10.63
p	94.38 ± 5.05△#	86.44 ± 12.07*#	73.71 ± 15.62*△
VAS at the last follow-up	0.29 ± 0.46#	0.56 ± 0.51	1.18 ± 1.01*

Data were presented as frequency count or means ± SD.

ANOVA = analysis of variance, L = plate-screw system fixation via L-shaped approach, LSD = Least-Significant Difference, S = plate-screw system fixation via sinus tarsi approach, S-S = Steinmann pin retractor-assisted reduction with the circular plate fixation via the sinus tarsi approach, SD = standard deviations.

*P* < .05 is considered to be statistically significant. The Chi-square test was used for counting data between groups. A comparison of measurement data between groups was performed using a one-way ANOVA, and LSD was used for multiple comparisons(

* vs. S-S group,

△ vs. S group,

# vs. L group).

**Figure 1.**
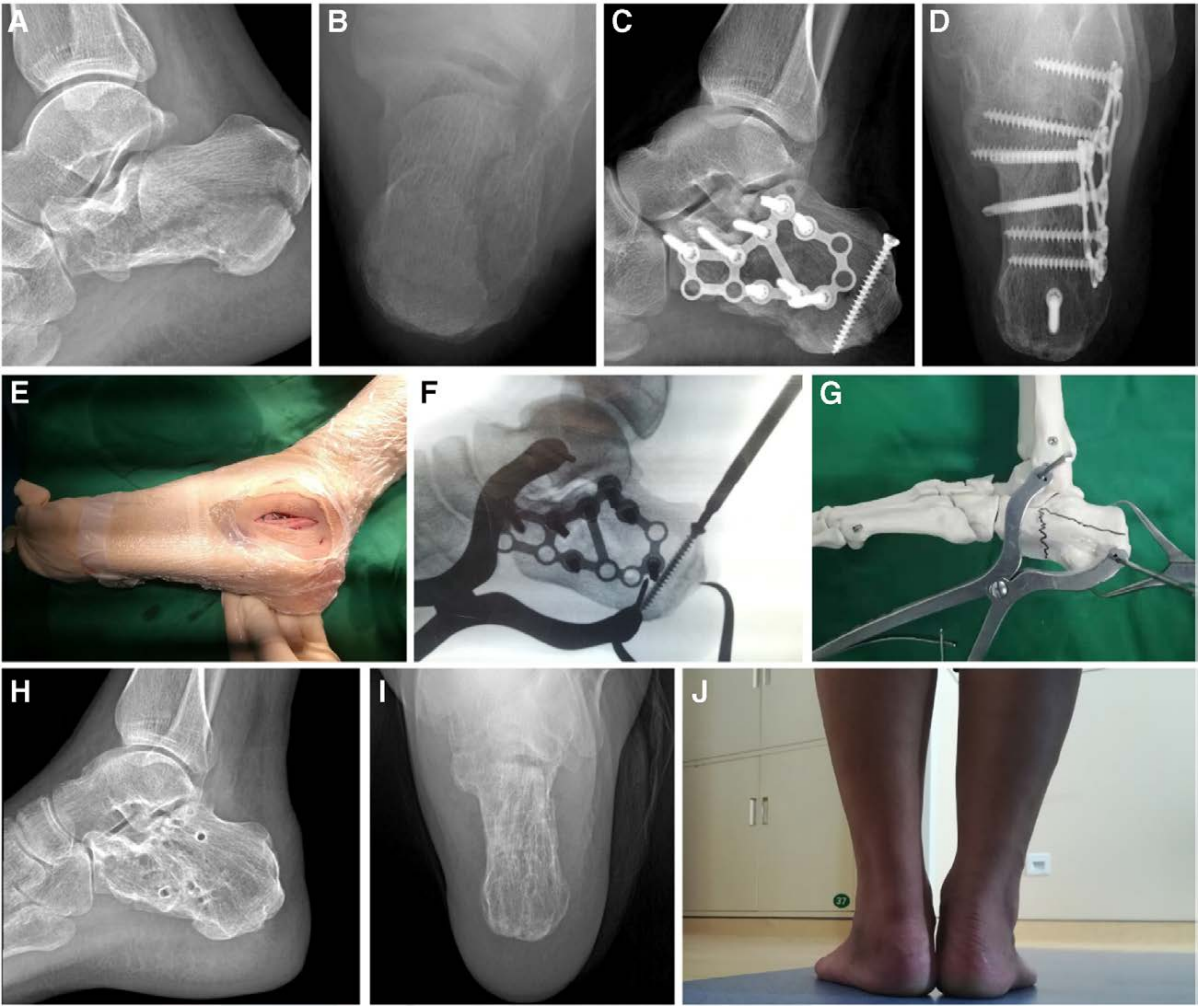
A 47-year-old male patient fell from a height of 3 meters. (A and B) Preoperative radiograph showing left calcaneal fracture (Sanders type IIB) with unnormal Böhler angle (–2°), height (82.7 mm), length (89.4 mm), and width (52.5 mm). (C and D) The postoperative radiograph showing Böhler angle (37°), height (91.6 mm), length (87.3 mm), and width (36.1 mm) are restored. (E, F and G) The protocol of Steinmann pin retractor-assisted reduction with circular plate fixation via sinus tarsi approach was used. (H and I) At the last follow-up, the radiological parameters were well maintained. (J) The patient had a normal alignment of the calcaneus and stable plantigrade foot, and the AOFAS score was 100. AOFAS = American Orthopedic Foot and Ankle Society.

**Figure 2.**
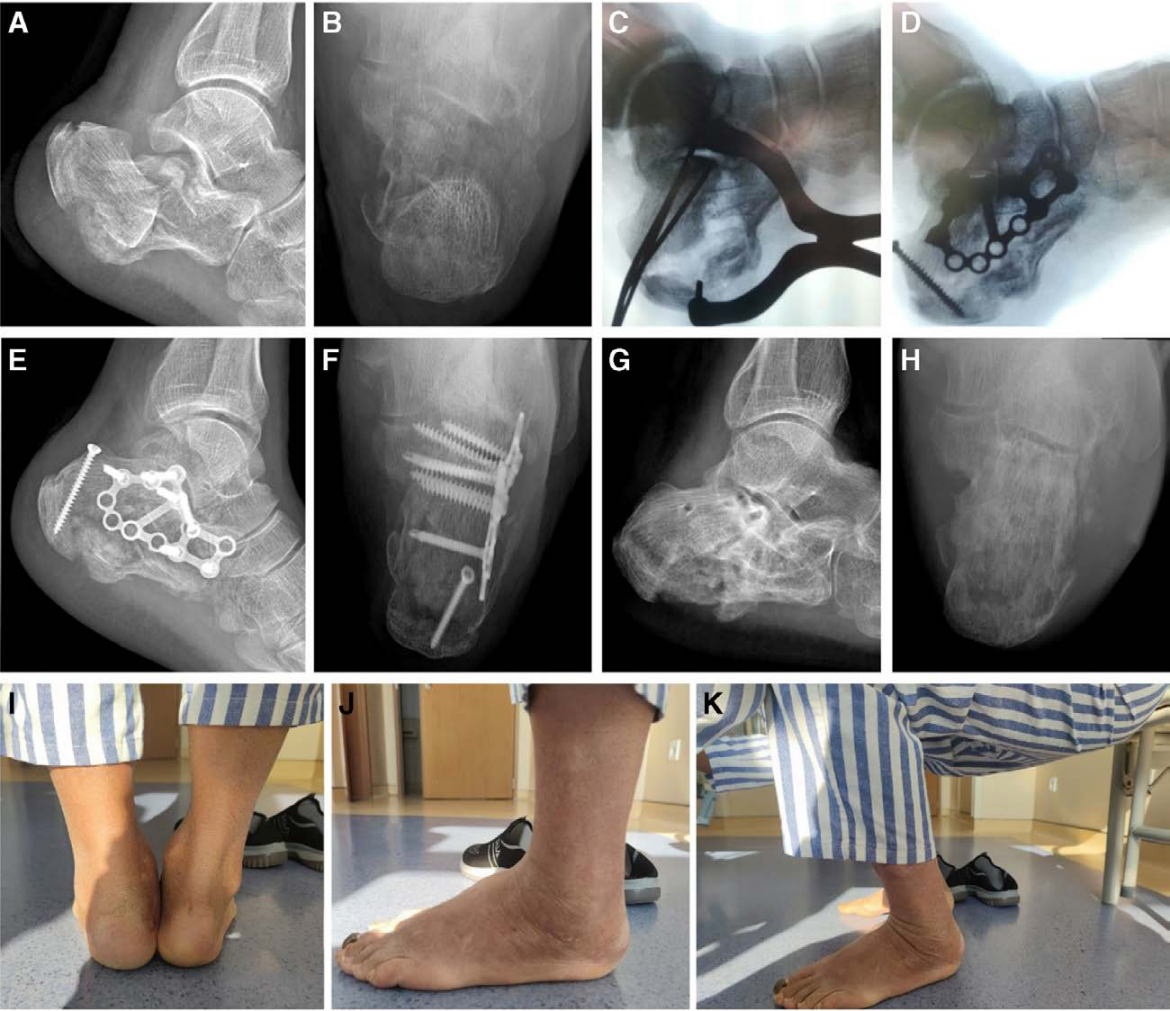
A 61-year-old male patient had a car accident. (A and B) Preoperative radiograph showing left calcaneal fracture (Sanders type ⅢAC, Tough type) with unnormal Böhler angle (–65°), height (91.8 mm), length (92.8 mm), and width (65.4 mm). (C and D) The protocol of Steinmann pin retractor-assisted reduction with circular plate fixation via the sinus tarsi approach was used, and the tuberosity fragment was fixed with a cancellous screw. (E and F) The postoperative radiograph showing the Böhler angle (32°), height (91.1 mm), length (89 mm), and width (39.9 mm) are restored. (G and H) At the last follow-up, the radiological parameters were well maintained. (I, J and K) The patient had a normal alignment of the calcaneus and stable plantigrade foot, and the AOFAS score was 96. AOFAS = American Orthopedic Foot and Ankle Society.

**Figure 3.**
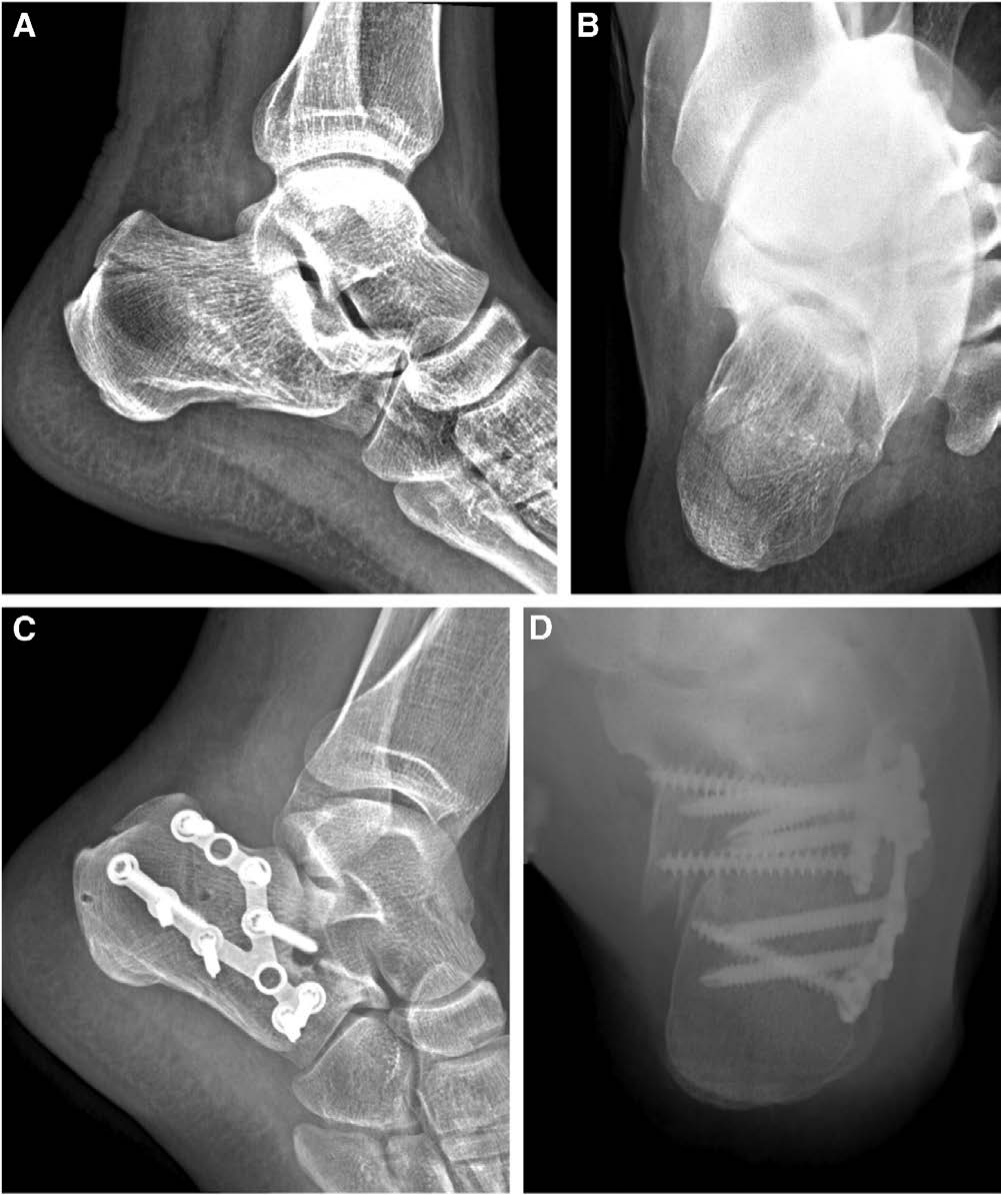
A 37-year-old male patient fell from a height of 1.5 m. (A and B) Preoperative radiograph showing left calcaneal fracture (Sanders type IIB) with unnormal Böhler angle (27°), Gissane angle (112°), height (81.4 mm), length (83.3 mm), and width (54.8 mm). 2-arm straight plate fixation via the sinus tarsi approach was used. (C) Postoperative lateral radiograph shoes well restoration of the Böhler angle (25°), Gissane angle (113°), height (86.7 mm), and length (82.3 mm). (D) However, the axial radiograph shows a broadening body (52.6 mm), accompanied by misalignment and varus deformity. AOFAS score was 82 at the last follow-up. AOFAS = American Orthopedic Foot and Ankle Society.

**Figure 4.**
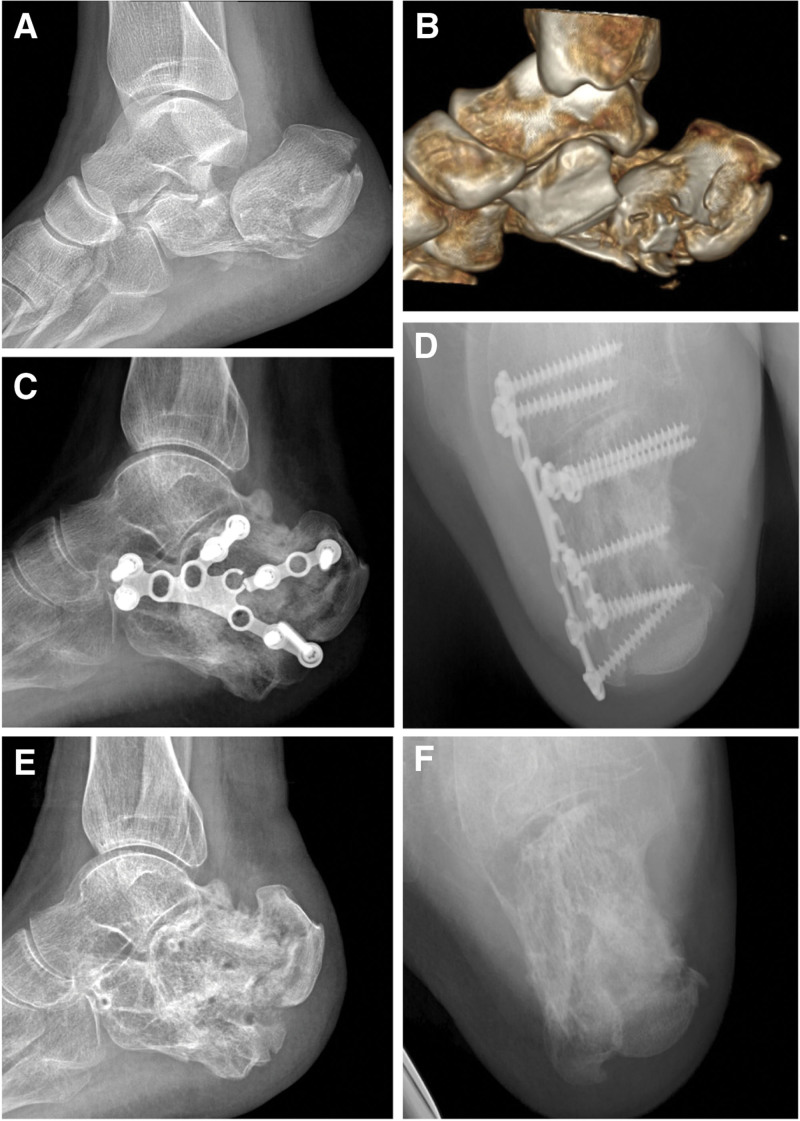
A 31-year-old male patient fell from a height of 3 meters. (A and B) A preoperative lateral radiograph and computed tomography (CT) image showing right calcaneus fracture (Sanders type ⅢAC, Tough type) with unnormal Böhler angle (–33°), Gissane angle (116°), height (82.4 mm), and length (83.4 mm). 3-ram straight plate fixation via the L-shaped approach was used. (C and D) At the last follow-up, the lateral and axial radiographs showed the Böhler angle (12°), Gissane angle (117°), height (82.5 mm), length (83.6 mm), and width (41.9 mm) are not well restored. (C, E and F) The patient experienced plate breakage and calcaneal malunion, and the AOFAS score was 49. AOFAS = American Orthopedic Foot and Ankle Society.

Patients in each group were operated on by a distinctly skilled surgeon who is specialized in one of the specified surgeries mentioned above. All patients underwent lateral, axial X-ray radiography (Fig. 1A and B, Fig. 2A and B, Fig. 3A and B, and Fig. 4A) and horizontal, coronal computed tomography (CT) scan. The three-dimensional reconstruction of the affected side was obtained before surgery (Fig. 4B). The surgery was performed after the wrinkle tested positive. The X-ray radiography of the unaffected side was obtained during the surgery by C-arm fluoroscopy. This was used as a reference for the reduction.

### 2.5. Surgical procedure

Surgery^[4]^ in the S-S group was performed under anesthesia in the prone position. Firstly, a sinus tarsi approach (Fig. 1E) was initiated with the posterior articular surface exposed. Secondly, two Steinmann pins were drilled into the talus and lateral process of calcaneal tuberosity respectively. The Steinmann pin retractor (Huatrau, Chinatrau instrument CO. Ltd, Guangzhou City, China) (Fig. 1F and G, Fig. 2C) was installed and gradually distracted the Steinmann pin until the length and height of calcaneus were restored. Thirdly, the step involving the posterior articular surface under direct vision was eliminated. Fourthly, the circular plate (Calcaneus Plates 2, Pure Titanium, 12 holes, Suzhou Kangli Orthopaedics instrument CO. Ltd, Suzhou, China) was fixed.

Surgery in the S or L group was performed under anesthesia in the prone or lateral position. The sinus tarsi approach was used in the S group. The prying method with a “joystick” without a retractor was used for the reduction process, and the circular or 2-arm straight plate was used for the fixation. The L-shaped approach was used in the L group. No Steinmann pin retractor was used. The H or 3-arm straight plate was used for the fixation after a satisfactory reduction was achieved. Finally, a drainage tube was fixed for each patient before closing the surgical openings.

### 2.6. Postoperative management and evaluation

All the patients were administered prophylactic antibiotics and non-steroidal anti-inflammatory drugs, elastic bandage dressing, and the same detumescence interventions postoperatively. Patients were encouraged to do toe flexion and dorsiflexion actively post-anesthesia. Surgery stitches were routinely removed two weeks postoperatively. Subsequently, a full-day time exercise for the subtalar joint was recommended. The unilateral injury was allowed for partial weight-bearing with crutches ten weeks postoperatively until the evidence of the excellent condition of bridging in the fracture site, and the absence of tapping pain along the axis of the calcaneus was observed. Full weight-bearing without crutches was allowed gradually over the next six weeks. However, the bilateral injuries needed additional two weeks for partial-bearing under the guidance of the therapist.

### 2.7. Follow up

The radiological parameters, including height (including talus), length, Böhler angle, and Gissanes angle in the lateral view, and the calcaneal body width in the axial view, were measured with the PACS (Picture Archiving and Communication Systems, version 2.5, Founder Group, Beijing, China) (Fig. 1C and D, Fig. 2C and D, Fig. 3E and F, Fig. 4C and D). Complications postoperatively, including incision complication, malunion, subtalar joint stiffness, traumatic arthritis, sinus tarsi syndrome, and compartment syndrome were recorded. The clinical evaluation involved a visual analog scale (VAS) (0 = no pain, 10 = maximum imaginable pain), and the American Orthopedic Foot and Ankle Society (AOFAS) Ankle-Hindfoot score was recorded at the last follow-up. Radiological parameters, complications, and clinical evaluation were carried out by an independent physician who was not involved in the surgeries.

### 2.8. Statistical analysis

Where applicable, data were presented as frequency count or means ± standard deviation. A comparison of paired data was performed using a paired *t*-test. The Chi-square test was used for counting data. A measurement data comparison was performed using a one-way analysis of variance (ANOVA), and least-significant difference (LSD) for multiple comparisons between groups. All statistical analyses were performed using the (IBM SPSS Statistics Version 19, SPSS 19.0). *P* values < 0.05 were considered to be statistically significant.

## 3. Results

Patients were followed up at least 6 months postoperatively, with no cases of sinus tarsi or compartment syndrome. Demographic and group information was presented in Table 1. There was no statistical difference in age, sex, affected side, injury mechanism, Essex-Lopresti type, Sanders type, accompanied fractures, smoking history, the time from trauma to operation, operation duration, and follow-up time among groups. Complications postoperatively were presented in Table 2, and there was no statistical difference among the groups. Two cases in the L group had wound edge necrosis, with one eventually degenerating into a wound infection. However, after an anti-infection therapy and regular incision dressing change, the infected wound healed 12 weeks postoperatively. One case in the S-S group had peroneus longus and brevis tendons injuries during the surgery. The tendons were sutured, and functioned normally at the last follow-up.

**Table 2:** Complications postoperative.

	S-S (20 patients)	S (17 patients)	L (15 patients)
Incision complication			
Wound edge necrosis (n)	0	0	2
Infection (n)	0	0	1
Hematoma (n)	0	0	0
Sural nerve injury (n)	1	1	2
Tendon injury	1	0	0
Malunion (Stephens and Saunders)			
Type Ⅰ (n)	1	3	1
Type Ⅱ (n)	1	1	3
Type Ⅲ (n)	0	0	1
Subtalar joint stiffness (n)	2	5	7
Traumatic arthritis (n)	1	1	4
Sinus tarsi syndrome (n)	0	0	0
Compartment syndrome (n)	0	0	0

Data were presented as frequency count.

L = plate-screw system fixation via L-shaped approach, S = plate-screw system fixation via sinus tarsi approach, S-S = Steinmann pin retractor-assisted reduction with the circular plate fixation via the sinus tarsi approach.

*P* < .05 is considered to be statistically significant. The Chi-square test was used for the comparison of the counting data between groups.

Few patients in each group experienced local skin numbness due to the sural nerve injury during surgery. Nevertheless, their quality of life was not disturbed by it. Patients had a normal alignment of the calcaneus and stable plantigrade foot without signs of axial deviation or chronic swelling, except 11 patients who experienced malunion at the last follow-up. There were screw loosening and implant breakage in a patient each in the S-S L group. The value of VAS in the S-S group was 0.29 ± 0.46 at the last follow-up and manifested statistically significant improvement in comparison with the L group. The AOFAS Ankle-Hindfoot score in the S-S group was 94.38 ± 5.05 at the last follow-up and also manifested significant improvements in comparison with the S and L groups (Table 1). In the S-S group, the Böhler angle (32.34 ± 3.56°), width (36.48 ± 3.91 mm), and height (88.87 ± 4.12 mm) (Table 3) of the calcaneus had significantly improved as well, (relative changes vs. preoperative: 34.38 ± 18.50°, –10.13 ± 6.98 mm, 8.75 ± 4.82 mm) (Table 4) in comparison with preoperative state, the S and L groups. These parameters (the Böhler angle: 31.76 ± 3.37°, width: 36.47 ± 3.72 mm, height: 87.23 ± 3.83 mm) (Table 3) were maintained at the last follow-up.

**Table 3:** Radiological parameters preoperative, postoperative, and the last follow-up.

Group	Böhler angle (°)			Gissane angle (°)			Calcaneal height (mm)			Calcaneal length (mm)			Calcaneal body width (mm)		
	Preoperative	Postoperative	Last follow-up	Preoperative	Postoperative	Last follow-up	Preoperative	Postoperative	Last follow-up	Preoperative	Postoperative	Last follow-up	Preoperative	Postoperative	Last follow-up
S-S	–2.14 ± 17.58	32.34 ± 3.56△#	31.76 ± 3.37△#	119.24 ± 15.41	118.05 ± 8.09	118.05 ± 8.16	80.11 ± 7.15	88.87 ± 4.12△#	87.23 ± 3.83△#	83.71 ± 4.97	86.69 ± 3.72	86.43 ± 3.78	46.61 ± 8.01	36.48 ± 3.91△#	36.47 ± 3.72△#
S	9.50 ± 11.65	25.50 ± 7.79*	23.50 ± 8.47*	119.44 ± 7.97	115.83 ± 7.71	116.72 ± 5.76	80.61 ± 5.27	84.35 ± 4.92*	82.06 ± 5.91*	83.50 ± 6.59	84.88 ± 6.21	83.99 ± 6.37	47.92 ± 7.78	43.37 ± 4.80*	42.77 ± 5.75*
L	5.41 ± 17.84	23.29 ± 6.56*	20.29 ± 6.74*	123.12 ± 9.29	122.47 ± 8.83	122.00 ± 6.03	80.62 ± 6.75	84.51 ± 6.23*	81.48 ± 5.60*	82.05 ± 5.01	84.19 ± 4.76	83.83 ± 4.01	45.38 ± 4.39	41.62 ± 3.98*	42.97 ± 4.70*

Data are presented as mean ± SD.

ANOVA = analysis of variance, L = plate-screw system fixation via L-shaped approach, LSD = Least-Significant Difference, S = plate-screw system fixation via sinus tarsi approach, S-S = Steinmann pin retractor-assisted reduction with the circular plate fixation via the sinus tarsi approach, SD = standard deviations.

*P* < .05 is considered statistically significant. A comparison of data between groups was performed using a *t*-test for paired data. A comparison of measurement data between groups was performed using a one-way ANOVA, and LSD was used for multiple comparisons (

* <0.05 vs. S-S group,

△ <0.05 vs. S group,

# <0.05 vs. L group).

**Table 4:** Relative changes of radiological parameters in different periods.

Grou	Postoperative vs. preoperative					Last follow-up vs. postoperative				
	Böhler angle (°)	Gissane angle (°)	Calcaneal height (mm)	Calcaneal length (mm)	Calcaneal body width (mm)	Böhler angle (°)	Gissane angle (°)	Calcaneal height (mm)	Calcaneal length (mm)	Calcaneal body width (mm)
S-S	34.38 ± 18.50△#	–0.95 ± 15.63	8.75 ± 4.82△#	3.12 ± 4.13	–10.13 ± 6.98△#	–0.48 ± 0.93	–0.24 ± 3.45	–1.64 ± 2.40	–0.26 ± 1.09	–0.01 ± 0.61
S	16.00 ± 13.67*	–3.61 ± 8.06	3.74 ± 3.50*	1.38 ± 2.65	–4.55 ± 7.14*	–2.00 ± 2.79	0.89 ± 6.62	–2.29 ± 2.54	–0.89 ± 2.10	–0.61 ± 1.91
L	17.88 ± 20.58*	–0.65 ± 11.89	3.88 ± 4.64*	2.14 ± 3.73	–3.76 ± 5.53*	–3.00 ± 5.71	–0.47 ± 7.07	–3.03 ± 3.42	–0.36 ± 2.37	1.35 ± 3.57

Data are presented as mean ± SD.

ANOVA = analysis of variance, L = plate-screw system fixation via L-shaped approach, LSD = Least-Significant Difference, S = plate-screw system fixation via sinus tarsi approach, S-S = Steinmann pin retractor-assisted reduction with the circular plate fixation via the sinus tarsi approach, SD = standard deviations.

*P* < .05 is considered statistically significant. A comparison of measurement data between groups was performed using a one-way ANOVA, and LSD was used for multiple comparisons (

*< 0.05 vs. S-S group,

△ <0.05 vs. S group,

# <0.05 vs. L group).

## 4. Discussion

Controversies between surgical and non-surgical management for the treatment of intra-articular calcaneal fractures are a long-standing issue.^[5]^ Recent literature^[6–10]^ reports that the surgical protocol has more benefits than non-surgical management. These are mainly reflective in radiological and clinical evaluations. The consensus^[11]^ for the indications of non-surgical management is extra-articular and non-displaced intra-articular fractures. The minimally invasive approach represented by the sinus tarsi approach has its advantage, however, it also has a downside of not fully exposing the calcaneal body, tuberosity, or sustentaculum tail. If there is an innovative method that could effectively provide a sound reduction, it could extensively promote the minimally invasive approach going further. However, till date few reports have made references to this. One study^[12]^ referred to the anterolateral fragment open-door technique via the sinus tarsi approach for the treatment of intra-articular calcaneal fractures. In response, we proposed a solution, which is the Steinmann pin retractor technique. To better elucidate this technique, comprehensive knowledge of calcaneal biomechanics is necessary.

The calcaneus has three articular surfaces to match the talus and cuboid bone respectively. The largest among them which is the posterior articular surface with a sagittal inclination contributes to the formation of Böhler and Gissane angles in the lateral view of X-ray, and bears most of the weight load. The trabeculae below the posterior surface extend to the tuberosity and anterior articular surface, forming the primary and secondary compressive trabeculae. The primary tensile trabeculae extend from the tuberosity to the anterior of the bone. Thus a stable triangular trabecular structure is formed. This is considered a real weight-bearing structure to the calcaneus,^[13]^ and helps to achieve a normal function of the “da Vinci`s bridge”.^[14]^ In reference to the intra-articular calcaneus fracture, the posterior articular surface was impacted into the body of the calcaneus, and the vertices of the load-bearing structure were destroyed. Restoring the Böhler angle and height of the calcaneus is to directly restore the vertices of the load-bearing structure and the triangular trabecular structure. With the assistance of the Steinmann pin retractor, adequate subtalar space was provided which facilitated restoring the Böhler angle and the height of the calcaneus. Besides that, the overlapped fracture fragments were separated and contributed to the restoration of the broadening body. The orientation of the calcaneus in the axial and sagittal planes is crucial to maintaining the longitudinal arch of the foot; the retractor aids in restoring this alignment via an insertion angle of the pin at the lateral process of calcaneal tuberosity.^[4]^ A patient in the S group who had a 2-arm straight plate used, experienced calcaneal malunion, pain, and subtalar joint stiffness at the last follow-up. The lateral X-ray image (Fig. 3C) showed a sound restoration of height, length, Gissane, and Böhler angles. Nevertheless, broadening, misalignment, and varus deformity was found in the axial image (Fig. 3D) because the overlapped fracture fragments obstructed the restoration of the broadening body without the retractor. For tongue-type fractures, according to the Essex-Lopresti classification,^[15,16]^ fixing the tuberosity fragment with a cancellous screw (TS) (Fig. 1F, Fig. 2D and F) is necessary, since the screws counteract the distraction generated by the Achilles tendon. At the last follow-up, a patient in the L group with a tongue-type fracture had calcaneal malunion (Fig. 4E and F). The 3-ram straight plate broke (Fig. 4C and D), and the fracture fragment was re-displaced. This can be avoided with the help of a tuberosity screw.

Compared with the L-shaped approach, the sinus tarsi approach can effectively reduce the incidence of incision complications.^[17]^ However, surgeons are still faced with other complications,^[18,19]^ such as infection, nerve or tendon injury, malunion, etc. Of all the complications, malunion is the toughest to deal with. The classification for malunion was described by Stephens and Saunders.^[20,21]^ Correcting malunion is difficult and the outcome is unpredictable.^[22]^ Dealing with malunion is a real challenge even for operative expertise and has gone beyond the scope of our study. Though we have not found a significant difference between these groups in complications postoperatively, the S-S group manifested a lower complication rate, either incision complications or malunion rate, which should be ascribed to the application of the Steinmann pin retractor.

## 5. Conclusion

Steinmann pin retractor effected a sound reduction. It also manifested radiological and clinical advantages over the S and L protocols. By reason of the aforementioned, it could be recommended as useful reduction equipment for the minimally invasive treatment of calcaneal fractures.

## Author contributions

**Conceptualization:** Bin Zhao, Rongxiu Bi.

**Data curation:** Wenqian Zhao, Rongxiu Bi.

**Formal analysis:** Wenqian Zhao, Chao Liu, Rongxiu Bi.

**Funding acquisition:** Bin Zhao.

**Investigation:** Bin Zhao, Wenqian Zhao, Chao Liu.

**Methodology:** Bin Zhao.

**Resources:** Wenqian Zhao.

**Resources and supervision:** Rongxiu Bi.

**Software:** Bin Zhao, Wenqian Zhao, Chao Liu.

**Writing – original draft:** Bin Zhao.

**Writing – review & editing:** Isaac Assan.

## Supplementary Material


