# Supplementary material for: Steinmann pin retractor: An auxiliary reduction equipment for the minimally invasive treatment of calcaneal fractures
Source: Medicine (Baltimore). 2022 Sep 30;101(39):e30847. doi: 10.1097/MD.0000000000030847 (PMC9524867; doi:10.1097/MD.0000000000030847)
Supplement: Supplementary file 1 [file medi-101-e30847-s001.pdf]

Supplementary Information (Ethical approval)

寿光市中医医院医学伦理委员会审查申请表

Medical ethics inspection form of shouguang hospital of traditional Chinese medicine

序号（由委员会统一填写）<br>（ID number）	20170301	项目科室<br>(Department)	骨伤二科（orthopedics）
项目名称<br>(project name)	克氏针撑开器联合环形钛板经由跗骨窦入路治疗跟骨关节内骨折的临床研究<br>(Kirschner wire retractor-assisted reduction with circle plate fixation via sinus tarsi approach for intra-articular calcaneal fractures.)	项目起止时间<br>(project starting and ending time)	2017. 03-2019. 03
项目类别<br>(category of project)	A. 新药物临床试验(Clinical trials of new drugs)<br>B. 新器械临床试验(Clinical trials of new devices)<br>C. 新技术应用(Clinical trials of new technology) √<br>D. 人体标本收集(Collection of human specimens)<br>E. 其他（请注明）(others)：		

申请人（项目负责人）简要信息(Introduction of the project leader)<br>赵斌，医学博士，主治医师，骨伤二科副主任。<br>Bin Zhao, M.D. Attending doctor of orthopedics, Deputy director of the second orthopedics.							
姓 名<br>(Name)	赵斌<br>(Bin Zhao)	性别<br>(Gender)	男 Male	学 位<br>Certificate	博士 M. D.	科 室<br>Department	骨伤二科<br>Orthopedics
办公电话<br>（Office phone）	18853691152	传真<br>(Fax)		移动电话<br>(Telephone)	18853691152	电子邮箱<br>(E-mail)	benjaminyan1@163.com
目前主要研究方向<br>(Major research direction)	跟骨骨折的微创治疗（Minimally invasive treatment for calcaneal fracture）						

经费来源(Project funding source)： ☐政府(Government) ☐基金会(Foundation) ☐公司(Company) ☐国际组织(International organization) ☐其他(Other) √

研究内容摘要：(Outline of research, methods, observational index, et. al)：

背景

跗骨窦切口在跟骨骨折手术治疗中已经广泛地应用，但微创切口不利于显露跟骨外侧壁。通过经皮穿针手法牵引的方式无法满足复位要求，也不利于维持复位状态。跟常规钛板相比，任何一种跟骨微创板不能同时满足稳定的三角固定、最大幅度的覆盖外侧壁的双重要求，进而潜在影响跟骨高度、长度及宽度的恢复。我们应用克氏针撑开器辅助复位、环形钛板经跗骨窦入路治疗跟骨关节内骨折，并对其进行影像学及临床疗效评价。

方法

自 2017 年 3 月至 2018 年 11 月，12 例患有闭合性跟骨关节内骨折的自愿患者接受克氏针撑开器辅助撑开、环形钛板经跗骨窦入路治疗，所有患者均接受影像学及临床疗效评价。

手术步骤

腰硬联合麻醉成功后，患者取俯卧位，患肢气囊止血带止血。首先，双手挤压跟骨内外侧壁，部分恢复跟骨宽度，标记跗骨窦切口位置，C 型臂透视下，自外踝尖内下缘至骰骨近端划线，以便于更准确的显露跟距关节面。第二步，沿标记线行长约 4-5cm 切口，锐性分离腓骨长短肌肌腱腱鞘，接下来，在距骨和跟骨结节部位打入两枚克氏针，克氏针撑开器缓慢撑开，直到患侧跟骨的长度和高度恢复至接近对侧水平，遇到舌形骨折时在撑开前先用巾钳固定跟骨结节分离骨块。第三步，去除跗骨窦内脂肪组织显露跟距关节面，适度过牵克氏针撑开器可以将跟距关节面牵开 3-5mm，以便于有足够的空间复位关节面，恢复贝角和 G 角后用克氏针临时固定。第四步，骨膜下剥离跟骨外侧壁，直至环形钛板可以顺利插入，接下来，先用松质骨螺钉三角固定，加压跟骨体恢复宽度，然后再依次拧入松质骨和锁定螺钉进行固定。最后拔出克氏针，放置引流管。

Background

Sinus tarsi approach has been widely used for intra-articular calcaneal fractures successfully, however, the minimal incision limited extensive lateral wall exposure. Under this circumstance, acquire or maintain an accurate reduction of the calcaneal body with manual Kirschner wire distraction percutaneously was difficult. Which in turn, affects the reduction of the fracture, including the Böhler angle, Gissanes angle, the length, height and width of the calcaneus potentially. In our study, we used the Kirschner wire retractor assisted reduction and used the circle plate for fixation via sinus tarsi approach, subsequently, assessed the radiological and clinical outcomes, and complications after surgery.

Methods

From March 2017 to November 2018, 12 patients with closed calcaneal fractures were treatment with Kirschner wire retractor-assisted reduction and circle plate fixation via sinus tarsi approach. All patients received both clinical and radiological evaluations.

Surgical procedure

Surgery was performed under spinal-epidural anesthesia with the prone position. Firstly, squeezed the body of the

calcaneus with hands to restore the width of calcaneus partially and marked the sinus tarsi incision from the lower margin of the tip of the lateral malleolus to the proximal cuboid precisely on the skin. Secondly, a 4-5cm incision was made accordingly after disinfecting and an electric pneumatic tourniquet(pressure, 40 KPa) was used during operation. Sharply dissected the sheath of peroneus longus and brevis tendons from the lateral wall and protected the sural nerve carefully. Subsequently, two Kirschner wires were drilled into talus and lateral process of calcaneal tuberosity respectively. Gradually distracted the Kirschner wire with calcaneus retractor(Huatrau, Chinatrau instrument CO˙. Ltd, Guangzhou city, China) until the length and height of calcaneus approximated to the unaffected side, while it should be noticed that using a pointed reduction clamp to maintain the major tubercular fracture blocks together before distraction was necessary, especially in tongue type fracture. Thirdly, remove the fatty tissue from the tarsal sinus which contributed to exposing the posterior calcaneal facets clearly. Over distracted the retractor in order to provide a 3-5mm gap of joint space, which contributed to restoring the integrity of posterior calcaneal facets precisely. Subsequently, the pry-poking method was used to restore the Böhler and Gissanes angle according to the unaffected side and maintained these angels with Kirschner wire fixation temporary. Fourthly, moderate subperiosteal dissection along the lateral wall was performed until the circle plate(Calcaneus Plates 2, Pure Titanium, 12 holes, Suzhou Kangli Orthopaedics instrument CO. Ltd, Suzhou, China) achieved well position under C-arm fluoroscopy. Screwed-in 3-5 cancellous bone screws triangulate, by the way restored the width of the calcaneus, subsequently, screwed-in locking screws or cancellous bone screws via the incision or percutaneously. In the end, removed the Kirschner wires and placed the drainage tube percutaneously before closing.

申请人（项目负责人）承诺 (Commitment of project leader) ：

以上所填内容均属实，如获批准，我将严格按照提供的方案进行研究，并遵守寿光市中医医院医学伦理委员会的相关规定。(I will carry out the research in strict accordance with the scheme provided and abide by the relevant regulations of the medical ethics committee of shouguang hospital of traditional Chinese medicine, accept the supervision and inspection of the committee at any time, and voluntarily accept the punishment if any infringement.)

申请人（项目负责人）签字(Signature): [illegible]　　日期(Data): 2017.03.01

申报科室领导意见（如果领导是研究者，请副主管签字）(Comments of the head of the department)：

同意（Approve）

科室领导签字(Signature): [illegible]　　日期(Data): 2017.03.01

医学伦理委员会审批意见 (Comments of medical ethics committee)：

同意（Approve）

主任委员（签章）(Signature): [illegible]　　日期(Data): 2017.03.01　医学伦理委员会（盖章）

Committee on Medical Ethics of the Shouguang hospital of traditional Chinese medicine

'Kirschner wire retractor-assisted reduction with circle plate fixation via sinus tarsi approach for intra-articular calcaneal fractures.'

This study was carried out in strict accordance with the recommendations in the Guide of 'Methods for ethical review of biomedical research involving humans(2016)' from the state health and family planning commission of the People`s Republic of China. This research was approved by the Committee on Medical Ethics of shouguang hospital of traditional Chinese medicine(Permit Number: 20170301). All the patients will sign the informed consent before the surgery.

Committee on Medical Ethics of shouguang hospital of traditional Chinese medicine

03/05/2017

Supplementary Information（informed consent for patient）

寿光市中医医院

右跟骨骨折克氏针撑开器辅助复位环形钛板经跗骨窦切口内固定术知情同意书

患者姓名 [redacted]	性别 男	年龄 43 岁	病历号 [redacted]

疾病介绍和治疗建议

医生已告知我患有右跟骨骨折，需要在腰硬联合麻醉下进行克氏针撑开器辅助复位环形钛板经跗骨窦切口内固定术手术。

手术潜在的风险和对策

本次手术治疗是我院开展的新技术项目内容，是潍坊市 2018 年科技发展计划立项项目（2018 年第 024 号，wfwsjs_2018_024），且该项目已经通过医院医疗伦理审查委员会审查。对于本项目的实施后的预期疗效和潜在风险将如下列举，请患者悉知，并酌情考虑是否接受本次手术治疗。

医生告知我手术可能发生的风险，有些不常见的风险可能没有在此列出，具体的治疗方式根据不同病人的情况会有所不同，医生告诉我可与我的医生讨论有关我手术的具体内容，如果我有特殊问题可与我的医生讨论。

1. 我理解任何手术麻醉都存在风险。
2. 我理解任何所用药物都可能产生副作用，包括轻度的恶心、皮疹等症状，严重的可以导致过敏性休克，甚至危及生命。
3. 我理解此次手术的有点以及存在的风险和局限性：

1）. 麻醉意外，严重者可致休克、甚至危及生命；
2）. 据以往文献报道跗骨窦切口可以明显降低切口并发症的发生率，克氏针撑开器辅助复位可以弥补跗骨窦切口暴露不足的短板，环形钛板固定可以提供稳定的三角支撑，恢复跟骨体的宽度。但术中需要行一定程度的外侧壁骨膜下剥离，存在一定的切口并发症风险；
3）. 术中损伤神经、血管及邻近器官，如运动神经损伤导致相应肌肉功能失支配，引起肢体功能障碍甚至残疾；皮神经损伤致相应部位麻木疼痛；血管损伤导致大出血，严重者可致休克，肢体缺血性挛缩甚至危及生命。
4）. 围手术期心、肺、脑血管意外出现。
5）. 脑出血或脑栓塞，神志不清，昏迷甚至死亡；
6）. 心律失常，心肌梗死，心力衰竭，血压降低，休克，甚至死亡；
7）. 肺栓塞，呼吸功能障碍，呼吸衰竭甚至死亡；
8）. 伤口并发症：出血、血肿、裂开、不愈合、感染，瘘管及窦道形成；伤口不愈合需要二期行植皮、皮瓣移植或延迟关闭等；
9）. 术中止血带及尿管发生并发症；
10）. 骨折固定意外情况，需要二次或多次行手术治疗；
11）. 骨折碎裂、移位较重，骨折对位对线不满意，骨折畸形愈合；
12）. 术后骨折再移位；
13）. 跟骨骨折处骨折不愈合；
14）. 内固定物松动或假体松动，断裂，异物反应，骨折延迟愈合或骨折不愈合；
15）. 术中内固定物或手术器械断裂不能取出而滞留于体内；
16）术后肢体功能恢复不满意，邻近关节僵硬，创伤性关节炎；
17）. 骨折碎裂较重，影响血供，并发跟骨坏死；
18）. 术后伤口感染，骨髓炎；

特殊风险或主要危险因素

我理解根据我个人的病情，我可能出现以下特殊并发症或风险：

1.切口皮缘坏死的可能；2.术后并发创伤性关节炎，关节疼痛，关节功能障碍的可能；3.术中发现骨缺损，需要植骨的可能；4.术后骨不连或延迟愈合的可能；5.术后钢板断裂、松动的可能；6.感染 7.关节炎缓解不明显 8.关节炎较重需行关节融合术治疗。

一旦发生上述风险和意外，医生会采取积极应对措施。

患者知情选择

- 我的医生已经告知我将要进行的手术方式、此次手术及术后可能发生的并发症和风险、可能存在的其它治疗方法并且解答了我关于此次手术的相关问题。
- 我同意在手术中医生可以根据我的病情对预定的手术方式做出调整。
- 我理解我的手术需要多位医生共同进行。
- 我并未得到手术百分之百成功的许诺。
- 我授权医师对手术切除的病变器官、组织或标本进行处置，包括病理学检查、细胞学检查和医疗废物处理等。

患者签名[redacted] 签名日期[redacted]

如果患者无法签署知情同意书，请其授权的亲属在此签名：

患者授权亲属签名________ 与患者关系______ 签名日期 ______年____月_____日

医生陈述

我已经告知患者将要进行的手术方式、此次手术及术后可能发生的并发症和风险、可能存在的其它治疗方法并且解答了患者关于此次手术的相关问题。

医生签名[redacted] 签名日期[redacted]

[illegible]

骨科手术（操作）知情同意书

姓名：[redacted]　　性别：男　　病历号：[redacted]

患者[redacted]因病住骨伤二科（新）病区 047 床。

术前（拟）诊断___左跟骨骨折______________

建议（拟）实施___切开复位内固定术____________________手术（操作）

由于目前医学科学技术水平的局限，尚难杜绝接受手术治疗的病人在手术中和术后可能发生以下列以外和并发症，现向患者或患者亲属说明：

1、麻醉意外，严重者致呼吸心跳停止可能。

2、术中术后出现多脏器官功能衰竭，内分泌功能严重紊乱可能。

3、术中损害周围血管、脊髓、神经、肌腱及其他组织器官，致严重后果可能。

4、术中术后出血、感染致休克、骨髓炎、关节炎等，经久不愈，需进一步处理可能。

5、术中依具体病变情况，可能改变手术方案。

6、术中术后并发骨折、脱位可能。

7、内固定物无法完整取出可能。

8、手术仅做姑息性治疗或仅提供很小康复机会可能。

9、术后伤口延迟愈合或不愈合，皮肤坏死，需进一步处理可能。

10、术后患者病情较术前可能出现进步、无改善、退步。

11、术后骨折脱位无法完全复位可能。

12、术后骨折延迟愈合或不愈合，需再次手术可能。

13、术后植入物（内固定或假体）松动断裂，需进一步处理可能，年轻人在关节置换术后若干年内，因某些已知或未知原因出现磨损、松动，需要关节翻修手术。

14、术后植入物（自体或异体或异种骨及其他组织）吸收，需进一步处理可能。

15、术后病情复发可能。

16、术后肿瘤转移可能。

17、异体骨、韧带、内固定物、假体等生物及人造植入物致排异反应、过敏反应可能。

18、术后疤痕挛缩，影响外观或功能可能。

19、手术无法一次性彻底解决问题，需多次手术可能。

20、手术可能出现反应性精神障碍、心脑血管意外、肺栓塞、应激性溃疡、肢体静脉栓塞、筋膜间室综合症等意外及并发症。

21、术后功能恢复情况不完全取决于手术，需配合理疗、康复锻炼等综合措施。

22、其他。（如目前通用的某些手术方法在若干年以后可能废弃不用，不应视为手术方案不当。）

[illegible]

[illegible]

[redacted]

医师签名 [illegible]

上述情况已明知，并表示完全理解，同意进行手术（操作），愿意承担各风险。

患者本人签名：[redacted]

或代理人签名：______________　　与患者的关系：______________

或单位负责人签名：______________　　与患者的关系：______________

工作单位：______________　　电话：______________

谈话及签名日期：20 年 8 月 4 日

注：除患者不具备完全民事行为能力外，不是患者本人签名者必须先签署授权委托书，由委托书上患者指定的代理人签名。

张永青

Supplementary Information (Authorization for the use of the right of portrait)

Authorization for the use of the right of portrait (for patients)

I authorize Shouguang hospital of traditional Chinese medicine and the licensee (Bin Zhao) to take photos, video, and record my personal voice. I authorize Shouguang hospital of traditional Chinese medicine and the licensee (Bin Zhao) to do the following for these photos, vides, and recording materials, as well as portraits, names, and voices involved in them.

1. ***The use of such materials includes reproduction, distribution, display, performance and derivative works.***
2. ***The use of the above materials may include synthesis or modification and may be published in any existing or future media channels, including but not limited to newspapers, television, radio, the internet, social media, etc.***
3. ***The above materials may be used for any purpose, regardless of time or place, including but not limited to education, commerce, advertising, publications, publicity, etc.***

I will not claim any remuneration for the above use of the above materials, and acknowledge that Shouguang hospital of traditional Chinese medicine and the licensee have the exclusive right to use the above photos, videos, audios materials and all derivatives thereof, I waive my right to review or license the above materials for use in any print or electronic publicity, I hereby agree that Shouguang hospital of traditional Chinese medicine and its licensee have full authority to handle all matters related to the above materials, including but not limited to defamation of reputation, invasion of privacy, moral violation, copyright infringement, etc.

The execution of this power of attorney indicates that there is no objection to all the above terms.

Signature: [redacted] *Time:* 2021.7.2

Address: [redacted]

Phone number: [redacted] *E-mail:*

(The following part is completed by Shouguang hospital of traditional Chinese medicine.)

Department:

Signature: [illegible]

Time: 2021.7.2

Authorization for the use of the right of portrait (for the journal)

As the copyright holder, I authorize the journal to do the following for these photos, and the portraits of my patients.

1. ***The use of such materials includes reproduction, distribution, display, performance and derivative works.***
2. ***The use of the above materials may include synthesis or modification and may be published in Springer Nature, including but not limited to publish the image under a CC BY open access license.***
3. ***The above materials may be used for any purpose, regardless of time or place, including but not limited to print and digital.***

I will not claim any remuneration for the above use of the above materials, and acknowledge that the journal has the exclusive right to use the above photos, the portraits of my patients and all derivatives thereof, I waive my right to review or license the above materials for use in any print or digital publicity, I hereby agree that the journal has full authority to handle all matters related to the above materials.

The execution of this power of attorney indicates that there is no objection to all the above terms.

Signature: 彭阳 *Time:2021-07-02*

Address:3353#, shengcheng street, shouguang city, Shandong province, China.

Phone number: *+8618853691152* *E-mail:benjaminyan1@163.com*
